# CD24 Is a Potential Immunotherapeutic Target for Mantle Cell Lymphoma

**DOI:** 10.3390/biomedicines10051175

**Published:** 2022-05-19

**Authors:** Jimena Álvarez Freile, Natasha Ustyanovska Avtenyuk, Macarena González Corrales, Harm Jan Lourens, Gerwin Huls, Tom van Meerten, Ewa Cendrowicz, Edwin Bremer

**Affiliations:** 1 Department of Hematology, University of Groningen, University Medical Center Groningen, Hanzeplein 1, 9713 GZ Groningen, The Netherlands; j.alvarez@umcg.nl (J.Á.F.); n.ustyanovska.avtenyuk@umcg.nl (N.U.A.); m.gonzalez.corrales@umcg.nl (M.G.C.); h.j.lourens@umcg.nl (H.J.L.); g.huls@umcg.nl (G.H.); t.van.meerten@umcg.nl (T.v.M.); 2 Mossakowski Medical Research Centre, Polish Academy of Sciences, 02-106 Warsaw, Poland

**Keywords:** mantle cell lymphoma, CD24, immunotherapy, immune checkpoint, phagocytosis

## Abstract

CD24 and its ligand Siglec-10 were described as an innate immune checkpoint in carcinoma. Here, we investigated this axis in B-cell lymphoma by assessing CD24 expression and evaluating pro-phagocytic effects of CD24 antibody treatment in comparison to hallmark immune checkpoint CD47. In mantle cell lymphoma (MCL) and follicular lymphoma patients, high mRNA expression of CD24 correlated with poor overall survival, whereas CD47 expression did not. Conversely, CD24 expression did not correlate with survival in diffuse large B-cell lymphoma (DLBCL), whereas CD47 did. CD24 was also highly expressed on MCL cell lines, where treatment with CD24 antibody clones SN3 or ML5 potently induced phagocytosis, with SN3 yielding >90% removal of MCL cells and triggering phagocytosis of primary patient-derived MCL cells by autologous macrophages. Treatment with CD24 mAb was superior to CD47 mAb in MCL and was comparable in magnitude to the effect observed in carcinoma lines. Reversely, CD24 mAb treatment was less effective than CD47 mAb treatment in DLBCL. Finally, phagocytic activity of clone SN3 appeared at least partly independent of antibody-dependent cellular phagocytosis (ADCP), suggesting CD24/Siglec-10 checkpoint activity, whereas clone ML5 solely induced ADCP. In conclusion, CD24 is an immunotherapeutic target of potential clinical relevance for MCL, but not DLBCL.

## 1. Introduction

In the past decade, immune checkpoint inhibitors that re-activate adaptive immunity have transformed the treatment paradigm, with long-term remissions in previously end-stage patients in various cancer types [1]. More recently, immune checkpoints on innate immune cells have also gained prominence as therapeutic targets [2]. Most notably, the “don’t eat me” CD47–signal regulatory protein alpha (SIRPα) signaling axis inhibits phagocytosis by innate effector cells such as macrophages, dendritic cells, and neutrophils [3,4]. Interruption of this axis, using either antibodies or ligand-based therapeutics induces phagocytosis of cancer cells and has prominent antitumor activity in vitro and in vivo [5,6,7]. Moreover, this treatment also drives antigen presentation and the development of T cell immunity in mouse models [8]. For B-cell lymphoma, a combination of CD47 antagonistic antibody with the standard-of-care CD20 antibody rituximab (RTX) yielded complete responses in relapsed and refractory patients [9].

Other innate immune checkpoints are also increasingly investigated as potential therapeutic targets, including the leukocyte immunoglobulin-like receptor 1 (LILRB1) [10] and LILRB2 [2,11] as well as the CD24–SIGLEC10 axis [12]. CD24 has been extensively studied in the context of cancer biology, with it being defined as a cancer stem cell marker in various malignancies, such as breast [13], pancreas [14], and ovarian carcinoma [15]. CD24-mediated signaling further promotes cell migration, invasion, and cell proliferation [16,17,18]. More recently, in a hallmark 2019 report, CD24 was also described as an innate immune checkpoint with apparent significance in several solid cancer types [19]. Specifically, CD24 relayed anti-phagocytic signals to phagocytes through its interaction with Siglec-10, a lectin expressed on tumor-associated macrophages (TAMs). Accordingly, CD24 blockade using a monoclonal antibody (mAb) induced macrophage-mediated phagocytosis of breast, ovarian, and pancreas cell lines in vitro and inhibited tumor growth of xenografted breast cancer cell line MCF-7 in an NSG mouse model [19]. Notably, high expression of CD24 has been associated with poor prognosis in several cancers [20,21,22,23], an association that might be partially related to its checkpoint function on innate anti-cancer immunity.

CD24 is a small, heavily glycosylated protein attached to the cell membrane by a glycosyl-phosphatidylinositol (GPI) anchor [24]. On the plasma membrane, CD24 localizes in lipid rafts [25] and, among others, regulates B-cell receptor localization. CD24 has been reported to interact with many ligands, including various selectins (P-, L-, and E-) [26,27], cell adhesion molecules (L1- and N-CAM), high mobility group box 1 (HMGB1), and Sialic-acid-binding immunoglobulin-like lectins (Siglec-5 and -10) [19,28]. Further, CD24 can mediate homotypic interactions [29], and Ab-mediated CD24 cross-linking on human B cells activates mitogen-activated protein kinases, suggesting that CD24 activates intracellular signaling events [30].

CD24 is physiologically expressed on human B cells and has been employed as a phenotypic marker for early-stage immature B cells [31]. CD24 is also reportedly expressed in various cancers, including non-Hodgkin B-cell lymphomas (NHLs) in which CD24 levels were elevated compared to healthy subjects [32]. Thus, CD24 may be an immune checkpoint relevant for B-NHL. Of note, certain types of NHLs such as mantle cell lymphoma (MCL) or follicular lymphoma (FL), retained CD24 expression in contrast to healthy counterparts [33], with MCL being a more aggressive lymphoma that comprises about 3% to 10% of total NHL cases [34]. MCL is currently not curable with conventional chemoimmunotherapy, and new treatments are urgently needed [35,36]. For diffuse large B-cell lymphoma (DLBCL), the most common type of NHL in adults [37], CD24 expression was also reported, although in this case, high expression of CD24 mRNA correlated with better response to R-CHOP in activated B cells (ABC)-DLBCL, a specific subtype of DLBCL patients [38]. These findings suggest that CD24 might play a different role in MCL or FL than DLBCL.

In this study, we investigated the role of CD24 as a therapeutic target preclinically using cell lines and primary patient-derived samples, with a particular focus on MCL and DLBCL, but also other B-cell lymphomas such as follicular lymphoma and Burkitt lymphoma. Further, the pro-phagocytic activity of CD24 antibody treatment was compared to that of CD47 antibody treatment, and the mechanism of action of CD24 targeted therapy was investigated.

## 2. Materials and Methods

### 2.1. Reagents

CD47 antibody (InhibRx, Ab.6.12 clone 2A1) with human IgG4 was produced by GenScript (Nanjing, China). Anti-CD24 (Clone SN3) was purchased from Novus Biologicals (LLC, Littleton, CO, USA). Anti-CD24 (Clone ML5), mouse anti-CD19 (Clone HIB19), anti-Siglec-10 mAb (Clone 5G6), anti-CD24-APC (Clone SN3), CD11b-Alexa Fluor 594 (M1/70), anti-CD45-BV421, anti-CD19-BV785, anti-Siglec-10-APC, and anti-CD14-PE were purchased from Biolegend (San Diego, CA, USA). Rituximab (RTX) was obtained from the Hematology Department of the University Medical Centre of Groningen (UMCG) (Groningen, The Netherlands). Penta·His-Alexa Fluor 647 antibody was obtained from Qiagen (Hilden, Germany). Recombinant human Siglec-10 protein (His-tagged) was obtained from Abcam (Cambridge, MA, USA). Goat F(ab’)_2_ anti-mouse IgG (Fc-domain specific) were purchased from Jackson Immunoresearch Laboratories (West Grove, PA, USA). CellTrace™ CFSE and CellTrace™ Violet were obtained from Thermo Fisher Scientific (Cleveland, OH, USA) and Incucyte^®^ Cytolight Rapid Red Dye for Live-Cell Cytoplasmic Labeling (Cytolight Red) from Sartorius (Göttingen, Germany). 1,10-dioctadecyl-3,3,30,30–tetramethylindodicarbocyanine (DiD) was obtained from Invitrogen and AnnexinV-FITC/APC from either Thermo Fisher Scientific or Immunotools (Friesoythe, Germany). Anti-CD47-APC, CD5-PE (clone LT1), CD11b-APC (clone MEM-174), CD11b-FITC, granulocyte-macrophage colony-stimulating factor (GM-CSF), macrophage-colony-stimulating factor (M-CSF), interferon-γ (IFN-γ), and interleukin 10 (IL-10) were purchased from Immunotools (Germany). Lipopolysaccharide (LPS) was purchased from Sigma-Aldrich (Merck, MO, USA) and TGF-β was from Peprotech (Thermo Scientific, London, UK).

### 2.2. Cell Lines and Culture Conditions

MCL cell lines UPN-1, HBL-2, JeKo-1, Rec-1, Granta-519, Maver-1, and Z-138; Burkitt lymphoma cells Daudi, Ramos, and Raji; and carcinoma cell lines HT-29, Colo-205, SK-BR-3, SK-OV-3, ES-2, BT-474, DLD-1 were obtained from American Type Culture Collection (Manassas, VA, USA). DLBCL cell lines U-2932, SU-DHL-4, SU-DHL6, SU-DHL-10, and SU-DHL-2 were obtained from Deutsche Sammlung from Microorganism und Zellkulturen, (Braunschweig, Germany) and ATCC. All cells were cultured according to the supplier’s recommendation either in RPMI or DMEM (Lonza, Biowhittaker BE12–604F and BE12–155F) supplemented with 10% fetal bovine serum (FBS, Gibco™ Fetal Bovine Serum, USA) at 37 °C in a humidified 5% CO_2_ atmosphere and were regularly tested for mycoplasma infections by PCR.

### 2.3. Primary Samples

Healthy peripheral blood samples were received as buffy coats from Sanquin, The Netherlands, under agreement number NVT0465.01. Waste material from MCL patients at the Universitair Medical Centre of Groningen (The Netherlands) was received from the Hematology Department. These samples came from newly diagnosed MCL patients.

### 2.4. Isolation of Immune Cells and MCL Blasts

Monocyte-derived macrophages were obtained from whole blood (WB). Firstly, peripheral blood mononuclear cells (PBMCs) were isolated by density gradient centrifugation with Lymphoprep^TM^ according to the manufacturer’s recommendations (STEMCELL Technologies, Vancouver, BC, Canada). PBMCs were seeded in a 6-well plate at a density of 2.5 × 10^6^ cells/mL in RPMI 10% FBS containing the M0 differentiation cytokines GM-CSF or M-CSF (50 ng/mL each) for 7 days. To generate M1-type macrophages, M0 cells were treated with LPS (100 ng/mL) and IFN-γ (20 ng/mL) for 1 day. For M2c-type macrophages, M0 cells were primed with IL-10 (50 ng/mL) and TGF-β (50 ng/mL) for 2 days. Granulocytes were isolated from full blood samples of both healthy donors and patients from the pellet after density gradient centrifugation, followed by red blood cell (RBC) lysis using ammonium chloride lysis solution. Primary mantle cell lymphoma (MCL) blasts were obtained from peripheral blood of leukemic MCL patients and phenotyped using CD45/CD19/CD5 by flow cytometry. Autologous macrophages were obtained by isolation of monocytes from leukemic MCL PBMCs and differentiated into macrophages as described above.

### 2.5. Flow Cytometry Stainings

CD24 and CD47 expression was evaluated on a panel of MCL, DLBCL, BL, and carcinoma cell lines. For that, 5 × 10^4^ cells were incubated with FcR blocking reagent (IVIG, Nanogam, Sanquin, The Netherlands) and stained with anti-CD24-APC (Clone SN3) or anti-CD47-APC for 30 min at 4 °C. The same protocol was used to perform further surface stainings. The expression levels of both CD24 and Siglec-10 were also evaluated on primary material. In this case, PBMCs from healthy donors and MCL patients were incubated with Fc blocker solution (Nanogam) and subsequently stained with anti-CD45-BV421, anti-CD19-BV785, anti-CD5-PE, anti-CD24-APC/anti- Siglec-10-APC for B cells and blasts, or anti-CD45-BV421, anti-CD19-BV785, anti-CD14-PE and anti-CD24-APC/anti-Siglec-10-APC for monocytes. The percentage of CD24 and Siglec-10-positive cells within CD45+CD19+(CD5+) (B cells (blasts)) and CD45+CD19-CD14+ (monocytes) was analyzed. 

### 2.6. Macrophage-Mediated Phagocytosis Assay 

Flow cytometry-based phagocytosis assays were performed by co-culturing cancer cells and macrophages in RPMI 10% FCS, at an effector:target (E:T) ratio of 1:3 (for MCL and DLBCL) or 1:1 (for carcinoma cell lines) for 2 h in a humidified 5% CO_2_ incubator at 37 °C. Donor-derived macrophages were harvested from plates using TrypLE Express (Life Technologies, Carlsbad, CA, USA) prior to the co-culture. Cell lines were fluorescently labeled with Incucyte red (IR) or cell trace violet (CTV) according to the manufacturer’s instructions. Where indicated, cancer cells were pretreated with 1 μg/mL (MCL and DLBCL) or 10 μg/mL (carcinomas) of mouse IgG1 anti-CD24 (CD24-mIgG1) (Clone SN3), anti-CD24 (Clone ML5) or anti-CD47 (Clone 5F9-G4) antibodies, 1–10 ng/mL of rituximab (Truxima), 10 μg/mL recombinant human Siglec-10 (rh Siglec-10) protein (Abcam), 10 μg/mL of mouse IgG1 anti-CD19 (Clone HIB19) and the same concentrations of appropriate isotype controls. For some experiments, macrophages were pretreated with 100 μg/mL of Fc blocker solution (IVIG, nanogam, Sanquin) for 40 min at 4 °C or 10 μg/mL of anti-Siglec-10 mAb (Clone 5G6, Biolegend). After co-culture, phagocytosis samples were stained with anti-CD11b-FITC or -APC (Immunotools) to identify human macrophages. Samples were analyzed by flow cytometry (CytoFLEX, Beckman Coulter, Fullerton, CA, USA) and data were analyzed in the CytExpert Software (Beckman Coulter). Phagocytosis was measured as the number of CD11b+/CTV+ or IR+ macrophages, quantified as a percentage of the total CD11b+ macrophages. The total number of remaining cancer cells was counted using flow cytometry and counting beads (Biolegend). Each phagocytosis assay (independent donor and experimental group) was performed in a minimum of technical triplicate.

For the live-cell microscopy-based phagocytosis assay, M2c macrophages (2 × 10^4^ cells/well) were pre-seeded in 96-well plates for 24–48 h and IR or CFSE-labeled cancer cells were subsequently added to an effector:target (E:T) ratio of 1:3 and incubated for 2–3 h at 37 °C. Cancer cells were pretreated with anti-CD24 (SN3) or anti-CD47 (InhibRx) before co-culture. Subsequently, non-adherent MCL cells were removed by gently washing twice with RPMI 10% FCS. Cancer cell removal was qualitatively assessed by EVOS imaging (Thermo Fisher). For microscopy analysis, macrophages were stained with anti-CD11b Alexa Fluor-594 or anti-CD11b-FITC. Phagocytic uptake was assessed using IncuCyte S3^®^ live cell imaging system (Sartorius, Göttingen, Germany). Assays were repeated with a minimum of three independent donors to obtain experimental replicates. 

### 2.7. Flow Cytometry-Based Trogocytosis Assay

PMN-mediated trogocytosis assays were performed by co-incubating target cells and PMNs in RPMI 10% FCS at a ratio of 1:1 overnight in a humidified, 5% CO_2_ incubator at 37 °C. Tumor cells were pre-stained with 1,10-dioctadecyl-3,3,30,30–tetramethylindocarbocyanine (DiD) following the manufacturer’s instructions and pre-treated with 1 μg/mL of CD24 antibody (Clone SN3) and CD47 (Clone 5F9-G4, InhibRx, La Jolla, CA, USA) before co-culturing with PMNs. Assays were measured by flow cytometry (CytoFLEX, Beckman Coulter, Brea, CA, USA). The PMN population was gated based on SSC vs. FSC and the percentage of DiD+ PMNs was evaluated in the CytExpert Software (Beckman Coulter).

### 2.8. Generation and Validation of F(ab’)_2_ Fragments

SN3 F(ab’)_2_ was prepared using the Pierce™ Mouse IgG1 Fab and F(ab’)_2_ Micro Preparation Kit (Thermo Fisher Scientific). F(ab’)_2_ concentration was measured with a NanoDrop spectrophotometer (Thermo Fisher Scientific), and size was confirmed using non-reducing SDS-PAGE and Western blot. Alternatively to F(ab’)_2_ fragments, the Fc-domain of full anti-CD24-mIgG1 (Clone SN3) was blocked with goat F(ab’)_2_ anti-mouse IgG1 (Fc-domain specific). For that, the full antibody was incubated with goat F(ab’)_2_ in a 1:5 molar ratio for 30 min at RT. A volume of the mix corresponding to a final concentration of 1 μg/mL of full antibody was used in phagocytosis assays.

### 2.9. mRNA and Survival Data Analysis 

CD24 and CD47 mRNA expression data in different B-cell lymphomas were obtained from Chun et al. [39]. MCL (*n* = 71) survival data were obtained from Blenk et al. [40], FL (*n* = 77) data from Glas et al. [41], and BL (*n* = 41) from Sandeep et al. [42]. All datasets are available at the Precog Stanford database (https://precog.stanford.edu/). Previous treatments were: MCL patients received multiagent chemotherapy or no treatment; 36/71 FL patients received no treatment, 8/71 received radiotherapy (RT), 9/31 chlorambucil (chloramb), and the rest combinatory treatment including, for example, CHOP or cyclophosphamide (CVP). BL patients were treated with both CHOP-like regimes or intensive regimes, including high-dose cytarabine, CVP, or methotrexate. All patients, regardless of the treatment, were selected for the analysis, and the highest mean and distribution probe was used. An in-house-produced DLBCL transcriptome dataset comprising 1017 clinically annotated DLBCL patients was used [43]. Data were analyzed on the UCSC Xena platform (University of California, Santa Cruz, CA, USA), filtering the samples by R-CHOP treatment and highest distribution probe. Individual analysis for the major molecular subtypes of DLBCL, activated B cell (ABC), and germinal center B cell (GCB) was also performed. 

### 2.10. Statistical Analysis

The effect of the different antibodies on phagocytosis performed by different donors was determined by paired Student’s *t*-tests. The correlation between CD24 expression and phagocytosis levels upon CD24 antibody treatment was evaluated through a Pearson correlation test. A comparison of three or more variables was analyzed with a one-way ANOVA followed by the post hoc Tukey test. A comparison of survival plots was performed with a log-rank (Mantel–Cox) test. All tests were performed using GraphPad Prism (GraphPad Prism; GraphPad Software, La Jolla, CA, USA). Where indicated, * = *p* < 0.05; ** = *p* < 0.01; *** = *p* < 0.001. 

## 3. Results

### 3.1. CD24 Is Expressed in Several B-Cell Lymphomas, Being Most Highly Expressed in MCL, Where It Correlates with Poor Prognosis in Contrast to Hallmark Immune Checkpoint CD47 

**Figure 1:**
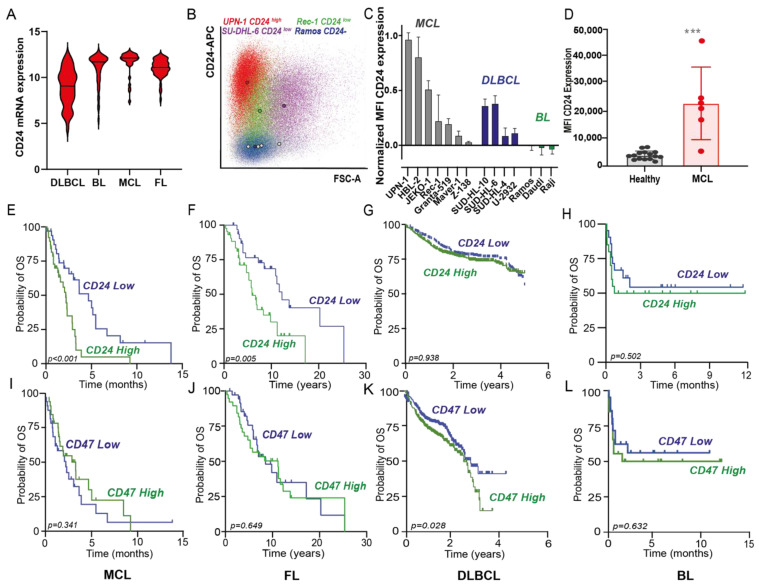
CD24 expression in several non-Hodgkin’s lymphomas (NHL) and correlation with survival. (**A**) CD24 mRNA expression (2log) in 4 subtypes of NHL, namely diffuse large B-cell lymphoma (DLBCL; *n* = 94), Burkitt lymphoma (BL; *n* = 58), mantle cell lymphoma (MCL; *n* = 42), and follicular lymphoma (FL; *n* = 64). (**B**) Dot-plot diagram of forward scatter vs. CD24 expression of representative cell line panel, illustrating high CD24 expression (UPN-1, red), low CD24 expression (Rec-1, green; SU-DHL-6, violet), or lack of CD24 expression (Ramos, blue) compared to isotype controls (gray). The colored circles indicate the mean value of the population. (**C**) Surface CD24 expression (mean ± SD of corrected MFI values, *n* = 3) in MCL, DLBCL and BL cell lines. Expression was normalized to the highest value, corresponding CD24 expression of UPN-1. (**D**) Surface CD24 expression (MFI values) of MCL blast cells (CD45+CD19+CD5+) (*n* = 6) and healthy donors (*n* = 13) (CD45+CD19+) cells. Mean ± SD, Student’s *t*-test, *** = *p* < 0.001 (**E***–***H**) Kaplan–Meier plots for CD24 and **(I***–***L**) CD47 expression in an MCL patient data set (*n*= 71) (**E**,**I**), an FL patient data set (*n* = 77) (**F**,**J**), a DLBCL patient data set (*n* = 1017) (**G**,**K**), and BL patient data set (*n* = 41) (**H**,**L**); log-rank test performed in all cases, with *p* < 0.05 considered significant.

CD24 is a well-established marker overexpressed in various carcinomas, where it is associated with poor survival. Furthermore, CD24 is a B lineage marker that is also expressed at the mRNA level in several types of NHL, such as MCL, FL, BL, and DLBCL, albeit with a large range of expression (Figure 1A). In line with this large expression range of mRNA in NHL, surface expression of CD24 in a panel of B cell lines composed of MCL, DLBCL, and BL ranged from high to negative (Figure 1B), with CD24 expression being the highest in the MCL cell lines UPN-1 and HBL-2 (Figure 1C). In DLBCL, SU-DHL-10 and SU-DHL-6 expressed the highest levels of CD24, although only at similar levels to MCL cell lines with low CD24 expression (e.g., Rec-1) (Figure 1B,C). Surface expression of CD24 was not found in any of the BL cell lines (Figure 1C). In line with this, primary patient-derived leukemic MCL cells (defined as CD45^+^CD19^+^CD5^+^) expressed elevated surface levels of CD24 compared to B cells from healthy donors (CD45^+^CD19^+^) (Figure 1D).

High mRNA expression of CD24 also significantly correlated with poor overall survival (OS) in MCL and FL compared to patients with low CD24 expression (Figure 1E,F). In contrast, CD24 did not correlate with OS in DLBCL patients treated with R-CHOP (Figure 1G), nor in separate cohorts of ABC or GCB DLBCL subtypes (Supplementary Materials Figure S1A,B) or BL (Figure 1H). Unlike CD24, mRNA expression levels of CD47 were similarly high in all lymphomas (Supplementary Materials Figure S1C). Further, the level of CD47 did not correlate with OS in MCL (Figure 1I), FL (Figure 1J), and BL (Figure 1L), but high expression of CD47 did negatively correlate with OS in DLBCL (Figure 1K) as previously reported by us [43]. Therefore, CD24 might play a significant role in certain hematological malignancies, such as MCL and FL, in which the well-known immune checkpoint CD47 may be less relevant.

### 3.2. CD24 Is a Target for Reactivation of Phagocytosis in MCL, with a Superior Effect Than CD47 Antibody Treatment

**Figure 2:**
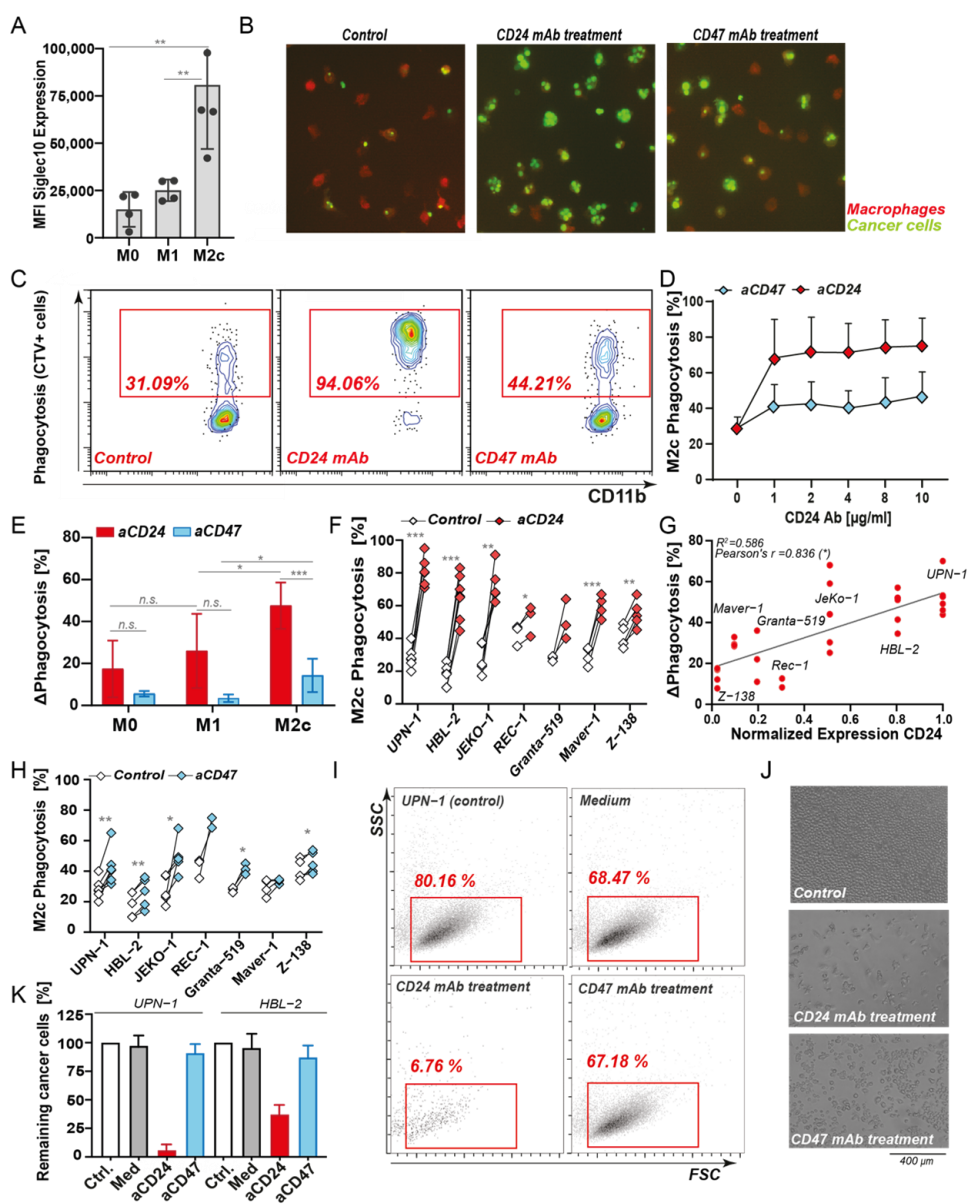
Phagocytic uptake and clearance of MCL cell lines upon CD24 and CD47 antibody treatment. (**A**) Levels of Siglec10 expression in M0, M1, and M2c type macrophages. (**B**) Microscopy images of macrophage-mediated phagocytosis of UPN-1 cells in M2c mixed culture (control, left), CD24 antibody SN3 (middle) or CD47 mAb InhibRx treatment (right), with macrophages in red (CD11b) and cancer cells in green (CSFE). (**C**) Flow cytometry analysis of UPN-1 phagocytosis with cell trace violet (CTV)-stained cancer cells and macrophages stained with CD11b-APC. Level of phagocytosis was determined as the percentage of CD11b+CTV+ cells. (**D**) Dose range of CD24 antibody SN3 and CD47 antibody InhibRx treatment in UPN-1 cells. (**E**) Phagocytosis levels of UPN-1 cells by M0, M1, and M2c-polarized macrophages upon SN3 and InhibRx treatment. (**F**) Percentage of phagocytosis upon CD24 SN3 antibody treatment vs. medium control of several MCL cell lines by M2c macrophages (of at least 3 independent donors). (**G**) Correlation between CD24 expression and increase in phagocytosis upon CD24 SN3 treatment in MCL cell lines. CD24 expression was normalized to the highest value, corresponding to UPN-1 CD24 expression. Pearson’s *r* = 0.836 (*) and linear regression *p* < 0.05. (**H**) Percentage of phagocytosis upon CD47 InhibRx treatment vs. medium control by M2c macrophages (of at least 3 independent donors) in several MCL cell lines. (**I**) Flow cytometry diagram showing the percentage of remaining UPN1 cell line after 2 h phagocytosis. (**J**) Bright-field microscopy pictures showing remaining UPN-1 cells after o/n co-culture with macrophages. (**K**) Percentage of remaining UPN-1 and HBL-2 cells in different experimental conditions (cancer cells only (Ctrl), mixed culture with medium control (Med), CD24 antibody SN3, and CD47 antibody InhibRx. All averaged values represent the mean ± SD of at least 3 independent experiments. Unless specified otherwise, statistics were performed using a two-sided paired Student’s *t*-test. ns: not significant; * *p* < 0.05; ** *p* < 0.01; *** *p* < 0.001.

To delineate the potential role of CD24 as a “don’t eat me” signal and therapeutic target for B-NHL, mixed macrophage/cancer cell cultures were subjected to CD24 antibody treatment. Hereto, firstly MCL was selected based on the high CD24 expression and association with survival. Secondly, M2c-differentiated macrophages were selected as these expressed higher levels of the CD24 ligand Siglec-10 than M1 macrophages (Figure 2A). Using confocal fluorescence microscopy, single fluorescently labeled and CD24-positive UPN-1 cells were clearly detected inside M2c macrophages after 3 h of co-culture in control conditions (Figure 2B, left panel). Upon treatment with CD24-mIgG1 antibody (clone SN3), prominent phagocytosis of cancer cells by M2c macrophages was detected with engulfment of several cancer cells per single macrophage in some donors (Figure 2B, middle panel). Similar treatment with CD47 blocking antibody InhibRx, as the ‘benchmark’ innate immune checkpoint, did trigger a clear increase in phagocytosis compared to untreated conditions, but the number of macrophages with engulfed cancer cells and the number of cancer cells per macrophage were lower than upon CD24 antibody treatment (Figure 2B, right panel). Subsequent quantification of phagocytosis using flow cytometry confirmed these findings, with ~30% phagocytosis upon control treatment that was increased to ~95% phagocytosis upon CD24 antibody treatment (Figure 2C), an increase similar to that upon quantification of microscopy data (see Supplementary Materials Figure S2A). In contrast, treatment with CD47 antibody only increased phagocytosis to ~45% (Figure 2C). The level of phagocytosis induction upon CD24 antibody treatment was already maximal at the dose of 1 µg/mL (Figure 2D, red diamonds). Treatment with CD47 antibody increased phagocytosis only by 15–20% compared to medium control and also reached its maximal level at 1 µg/mL (Figure 2D, blue diamonds). In line with expectations, treatment with CD24 mAb SN3 induced lower levels of phagocytosis of UPN1 cells by M1 macrophages, although InhibRx similarly induced a lower level of phagocytosis in M1 macrophages (Figure 2E).

The impact of CD24 antibody treatment in MCL was further evaluated using a panel of seven cell lines with different CD24 expression levels (Figure 1C), ranging from high (CD24^high^; UPN-1, HBL-2), moderate (CD24^mod^; JeKo-1) to low (CD24^low^; Rec-1, Granta-519, Maver-1, Z-138). Notably, the basal level of phagocytosis detected for these cell lines did not significantly correlate with CD24 expression (Supplementary Figure S2B). Nevertheless, treatment of the cells with CD24 mAb SN3 increased phagocytosis in CD24^high^ cell lines by up to 50–60%, in CD24^mod^ by ~45%, and in CD24^low^ cell lines by 10–30% (Figure 2F), with a strong positive correlation of CD24 expression with antibody-induced phagocytosis (Figure 2G, Pearson’s r = 0.836). In contrast, treatment with CD47 mAb InhibRx increased phagocytosis by ~20–25% for all of the MCL cell lines (Figure 2H), with all cell lines having consistently high expression of CD47 and no correlation between expression and phagocytosis being detected (Supplementary Figure S2C,D). Importantly, treatment with mouse IgG1 isotype control did not induce phagocytosis of CD24^high^ cell lines (Supplementary Figure S2E), nor did treatment of tumor cells alone with CD24 mAb induce apoptosis as measured by phosphatidylserine (PS) exposure [44], even upon cross-linking with a secondary anti-mouse antibody, within the time frame of the phagocytosis experiment (Supplementary Figure S2F).

When remaining cancer cells were counted in phagocytosis assays after 2 h, treatment with CD24 mAb SN3 almost completely removed UPN-1 cells from the solution, with an ~93% removal rate (Figure 2I). This observation is in agreement with the high level of engulfment observed visually after short-term incubation (Figure 2B,C) and the reduced cell numbers using bright-field microscopy after overnight culture (Figure 2J). Indeed, quantification of remaining cells using counting beads after 2 h of treatment yielded a high and significant reduction in tumor cell number for both UPN-1 and HBL-2 (Figure 2K). In contrast, CD47 InhibRx treatment did not significantly reduce cell number, neither after short term nor after 24 h incubation (Figure 2I–K, Supplementary Video S1). Thus, treatment of MCL cell lines with antibody-based CD24 targeting triggers extensive phagocytosis and a rapid decrease in cancer cell number in vitro.

### 3.3. CD24 mAb Treatment Increased Phagocytosis of Primary MCL Blasts by Autologous Macrophages and PMNs, but Did Not Induce High Level of Phagocytosis of Healthy Cells

**Figure 3:**
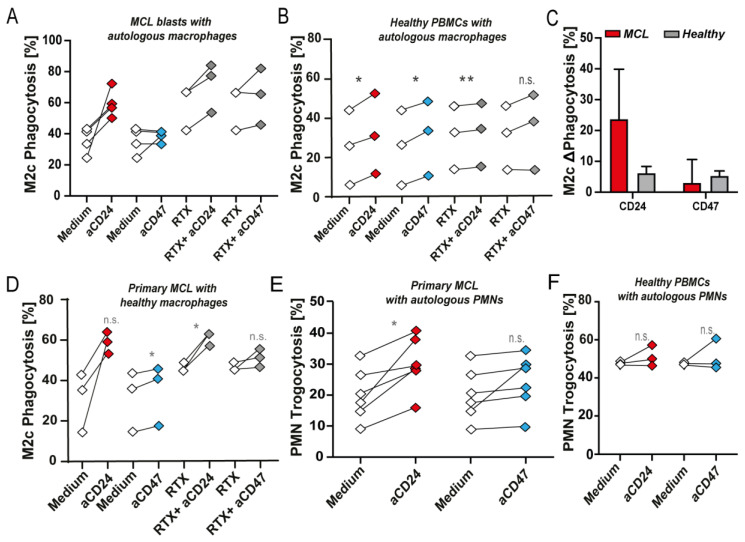
CD24 and CD47 antibody-mediated phagocytosis of MCL and healthy blasts by primary patient-derived autologous and allogeneic healthy donor macrophages. (**A**) Phagocytosis of MCL blasts by autologous macrophages upon CD24 mAb SN3 or CD47 mAb InhibRx treatment (*n* = 4) and combination treatment with RTX (*n* = 3). (**B**) Phagocytosis of healthy PBMCs by autologous macrophages upon CD24 mAb SN3 or CD47 mAb InhibRx treatment and in combination with RTX. (*n* = 3). (**C**) Comparison between phagocytosis levels induced upon CD24 and CD47 antibody treatment of MCL blasts (red) and healthy PBMCs (gray). (**D**) Phagocytosis of MCL blasts co-cultured with macrophages from healthy donors upon treatment with CD24 mAb SN3 or CD47 mAb InhibRx and in combination with RTX. (*n* = 3). (**E**) Trogocytosis of MCL blasts by autologous PMNs upon treatment with CD24 mAb SN3 or CD47 mAb InhibRx (*n* = 5). Statistical testing was performed using paired Student’s *t*-test. (**F**) Trogocytosis of healthy PBMCs by autologous PMNs upon treatment with CD24 mAb SN3 or CD47 mAb InhibRx (*n* = 3). Statistical testing was performed using paired Student’s *t*-test. Where indicated, n.s. = non-significant, * = *p* < 0.05; ** = *p* < 0.01.

Treatment of primary MCL blasts with CD24 mAb SN3 increased phagocytosis by autologous M2c macrophages by an average of 24%, whereas treatment with CD47 mAb InhibRx almost did not induce (3%) phagocytosis (Figure 3A). Further, the combination of CD24 mAb SN3 with RTX augmented RTX-mediated phagocytosis, whereas the combination with CD47 mAb InhibRx did not significantly potentiate activity (Figure 3A). When the same experiments were performed with PBMCs from healthy donors and autologous M2c macrophages, only an average of 6% increase in phagocytosis upon CD24 mAb treatment was observed, similar to CD47 mAb InhibRx (Figure 3B). Although not significant, CD24 antibody treatment clearly induced a higher phagocytic response in MCL compared to healthy samples (Figure 3C). In contrast, no significant difference was obtained for CD47 InhibRx treatment between MCL and healthy samples (Figure 3C). Combinatorial treatment with CD24 mAb SN3 and RTX did induce up to 5% phagocytosis of healthy PBMCs, but this effect was slightly lower than the combination of RTX and CD47 treatment (Figure 3B). In contrast, when primary MCL blasts were mixed with healthy donor-derived M2c macrophages, a similar potentiating effect to that obtained with primary autologous macrophages was detected (Figure 3D). Of note, this anti-cancer pro-phagocytic effect was also observed when primary patient-derived blasts were mixed with autologous PMNs, where SN3 treatment-induced up to 30% trogocytosis of primary PBMCs containing MCL blasts (Figure 3E). In line with this finding, Siglec-10 expression in PMNs was highly upregulated in MCL patients compared to healthy donors (Supplementary Materials Figure S3A). In contrast, InhibRx treatment did not significantly increase trogocytosis of MCL blasts (Figure 3E). Further, both CD24 and CD47 antibody treatment did not induce trogocytosis of healthy autologous PBMCs (Figure 3F). Thus, treatment of MCL with CD24 antibody-induced phagocytosis of CD24^+^ MCL cells alone and in combination with RTX, whereas this treatment minimally induced phagocytosis of healthy cells.

### 3.4. CD24 Antibody-Mediated Phagocytosis Is Superior to CD47 Checkpoint Targeting in MCL and Carcinoma, but Not in DLBCL

**Figure 4:**
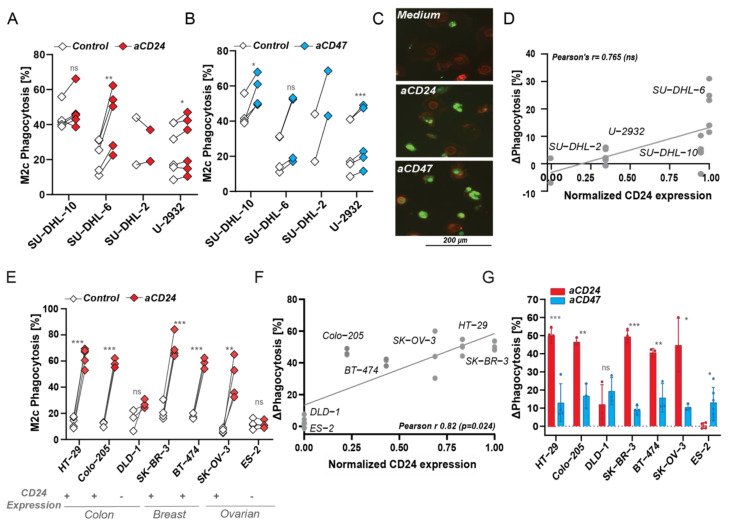
CD24 expression levels positively correlated with CD24 antibody-induced phagocytosis. (**A**) Percentage of phagocytosis upon CD24 SN3 antibody treatment vs. medium control by M2c macrophages (independent donors) in several DLBCL cell lines. Paired *t*-test. (**B**) Percentage of phagocytosis upon CD47 InhibRx antibody treatment vs. medium control by M2c macrophages (independent donors) in several DLBCL cell lines. Paired *t*-test. (**C**) Confocal microscopy images of phagocytosis of SU-DHL-6 where macrophages are in red and cancer cells are visible in green. (**D**) Correlation between CD24 expression and increase in phagocytosis upon CD24 SN3 treatment in DLBCL cell lines. CD24 expression was normalized to the highest value, corresponding to UPN-1 CD24 expression. Pearson’s *r* = 0.765 (ns) and linear regression *p* > 0.05. (**E**) Percentage of phagocytosis upon CD24 SN3 treatment vs. medium control by M2c macrophages (independent donors) in several colon (HT-29, Colo-205, DLD-1), breast (SK-BR-3, BT-474), and ovarian (SK-OV-3, ES-2) cell lines. Any effect was observed in CD24 negative cell lines (DLD-1, ES-2). Paired *t*-test. (**F**) Correlation between CD24 expression and increase in phagocytosis upon CD24 SN3 treatment in several colon (HT-29, Colo-205, DLD-1), breast (SK-BR-3, BT-474), and ovarian (SK-OV-3, ES2) cell lines. CD24 expression was normalized to the highest value, corresponding to SKBR3 CD24 expression. Pearson’s *r* = 0.82 (*) and linear regression *p* < 0.05. (**G**) Delta increase in phagocytosis compared to medium control upon CD24 and CD47 antibody treatment in several colon (HT-29, Colo-205, DLD-1), breast (SK-BR-3, BT-474), and ovarian (SK-OV-3, ES-2) cell lines. Student’s *t*-test. Where indicated, n.s. = non-significant, * = *p* < 0.05; ** = *p* < 0.01; *** = *p* < 0.001.

The prominent pro-phagocytic effect of CD24 but not CD47 antibody treatment on MCL cells suggested that CD24 is a potential novel target for therapy in MCL. However, targeting of the SIRPα/CD47 checkpoint did yield prominent clinical activity in a clinical trial in DLBCL and FL in combination with CD20 antibody rituximab. Notably, SN3 antibody treatment of DLBCL cell lines that expressed CD24, such as SU-DHL-10 and SU-DHL-6, did increase phagocytosis, albeit only up to 30% (Figure 4A)**.** Moreover, and in contrast to MCL, CD24 antibody treatment of the DLBCL cell line panel induced lower levels of phagocytosis than treatment with CD47 mAb InhibRx in most cell lines, with the exception of SU-DHL-6 (Figure 4B). Confocal fluorescence microscopy also revealed low levels of engulfment of SU-DHL-6 by M2c macrophages upon CD24 mAb SN3 treatment, whereas CD47 mAb InhibRx treatment led to slightly higher phagocytic uptake (Figure 4C). Of note, expression levels of CD24 in both SU-DHL-10 and SU-DHL-6 are similar to those found in CD24^low^ MCL cell lines (Figure 1C) and CD24 expression did not correlate with CD24 antibody-induced phagocytosis in DLBCL cell lines (Figure 4D). Thus, CD24 mAb treatment was inferior to CD47 mAb treatment in activating innate immune responses in DLBCL cell lines.

In contrast and in line with the literature, treatment with SN3 did significantly increase phagocytosis up to 50–60% in CD24^+^ carcinoma cells (Figure 4E, for expression data, check Supplementary Figure S4A). This increase in phagocytosis was again positively correlated with the levels of CD24 expression (Pearson’s *r* = 0.82) (Figure 4F), with CD24 expression of some carcinoma cell lines being higher than on UPN-1, the highest CD24-expressing MCL cell line tested. Similar to MCL cell lines and regardless of the high expression of CD47 (Supplementary Figure S4B), the specific increase in phagocytosis in carcinoma lines was significantly higher for CD24 than for CD47 antibody treatment for all CD24^+^ cells (Figure 4G). Thus, CD24 antibody treatment potentiated phagocytosis with greater effect in vitro than CD47 mAb treatment in MCL and carcinoma, but not in DLBCL.

### 3.5. Induction of Phagocytosis of CD24 Expressing Cells Is Only in Part an Effect of Breaking CD24-Siglec-10 ‘Don’t Eat Me’ Signaling

**Figure 5:**
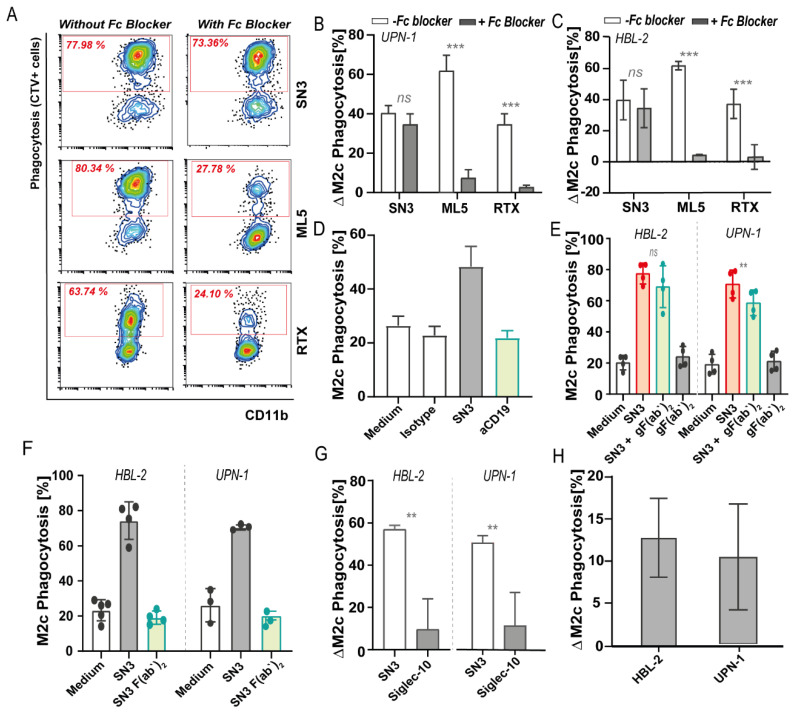
CD24 antibody treatment with two different murine anti-CD24 clones (SN3 and ML5) and Siglec-10 disruption of CD24/Siglec10 axis. (**A**) Flow cytometry diagrams showing the percentage of phagocytosis of UPN-1 cells upon CD24 antibody treatment with SN3 and ML5 clones by M2c macrophages in the presence or absence of high concentrations of Fc blocker. Rituximab (RTX) (anti-CD20) antibody was used as a control. (**B**) Increase in phagocytosis compared to medium control of UPN-1 cells upon CD24 antibody treatment with SN3 and ML5 antibody clones by M2c macrophages in the presence (gray) or absence (white) of high concentrations of Fc blocker. (**C**) Increase in phagocytosis compared to medium control of HBL-2 cells upon CD24 antibody treatment with SN3 and ML5 antibody clones by M2c macrophages in the presence (gray) or absence (white) of high concentrations of Fc blocker. *n* = 3, mean ± SD. *n* = 3, mean ± SD. Paired *t*-test, with *p* < 0.05 significant. (**D**) Phagocytosis of HBL-2 cells upon anti-CD24 (1 µg/mL, red) or anti-CD19 (10 µg/mL, green) treatment, being both mouse IgG1 antibodies. (**E**) Phagocytosis of HBL-2 and UPN-1 cells upon anti-CD24 treatment (SN3 clone) (red), same antibody pre-incubated with goat F(ab’)_2_ against mouse IgG Fc domain (gF(ab’)_2_) (green) or the goat F(ab’)_2_ alone (gray). Paired *t*-test, with *p* < 0.05 significant. (**F**) Phagocytosis levels of HBL-2 and UPN-1 cells upon CD24 SN3 treatment with the complete antibody (red) or F(ab’)2 preparations of the same antibody clone (SN3) (green). (**G**) Phagocytic uptake by M2c macrophages upon CD24 treatment with recombinant human Siglec-10 (His tagged) protein of HBL-2 and UPN-1 cells. (**H**) Increase in phagocytosis compared to medium control of HBL-2 and UPN-1 cells co-cultured with M2c macrophages previously incubated with 10 µg/mL of anti-Siglec-10 antibodies (5G6 clone). *n* = 3, mean ± SD. Where indicated, n.s. = non-significant, ** = *p* < 0.01; *** = *p* < 0.001.

In an effort to delineate the requisites and characteristics of CD24-based (re)activation of innate immunity, SN3 antibody clone (a mouse IgG1 binding to glycosylated epitope) and clone ML5 (a mouse IgG2a binding to the CD24 protein core) were first compared to their pro-phagocytic effect. Of note, both clones did effectively block the interaction of CD24 with recombinant Siglec-10 (Supplementary Materials Figure S5A). Both CD24 antibodies similarly induced phagocytosis of UPN-1 and HBL-2 cells, indicating the mAbs were functionally active irrespective of the epitope (Supplementary Materials Figure S5B). Nevertheless, the pro-phagocytic activity of mAb treatment may not only stem from the reported CD24/Siglec-10 checkpoint inhibition but may also stem from opsonization and induction of antibody-dependent cellular phagocytosis (ADCP). Indeed, when UPN-1 cells pretreated with mAbs were mixed with macrophages in the presence of high concentrations of Fc blocker to prevent ADCP-mediated activity, ML5-induced phagocytosis was abrogated (Figure 5A,B), similar to blocking effects observed for RTX (Figure 5B), a human IgG1 antibody that induces an ADCP [45]. Specifically, the delta increase in phagocytosis decreased from up to 60% without Fc blocker to <10% when Fc blocker solution was used for both UPN-1 and HBL-2 (Figure 5B,C). However, SN3-induced phagocytosis of UPN-1 was not significantly inhibited in the same setting (Figure 5A), with a non-significant <10% variation in the delta increase in phagocytosis upon inclusion of Fc blocker solution, for both UPN-1 and HBL-2 (Figure 5B,C). This data suggests that SN3 functions as a CD24 checkpoint inhibitor independent of functional mIgG1 activity, whereas ML5 activity is merely due to the opsonization of cancer cells, in agreement with its IgG2a backbone [46]. Indeed, mIgG1 is reported to not effectively trigger ADCP, with, e.g., also no phagocytosis detected upon treatment with CD19 mIgG1 antibody (Figure 5D). In line with this mode-of-action for SN3, the blocking of Fc-FcγR-dependent interaction using a goat F(ab’)_2_ anti-mouse IgG1 (Fc specific), as an alternate way to block SN3 IgG interaction with FcR (Supplementary Figure S5C), only decreased phagocytosis upon SN3 treatment by ~11% and ~9% for UPN-1 and HBL-2, respectively (Figure 5E). Thus, SN3 appears to have ADCP-independent activity reminiscent of checkpoint inhibition. However, in apparent contradiction, a direct F(ab’)_2_ preparation of SN3, generated by Ficin-mediated cleavage of the mIgG1 Fc (Supplementary Figure S5D), did not have significant pro-phagocytic effects on UPN-1 and HBL-2 (Figure 5F). Notably, the SN3 F(ab’)_2_ did block the binding of APC-labeled CD24 antibody to HBL-2 cells (Supplementary Figure S5E), indicating the preparation was functionally active. Further, the combination of SN3 F(ab’)_2_ with 1 ng/mL of RTX did not augment RTX-induced phagocytosis levels (Supplementary Figure S5F). Thus, although most evidence pointed to SN3-mediated innate checkpoint activity that was FcR-independent, conflicting results were obtained using SN3 F(ab’)_2_.

Previously, checkpoint activity of CD24 targeting by SN3 was suggested to derive from blocking its interaction with Siglec-10 on immune effector cells. Although tumor-associated macrophages could not be directly assessed due to lack of material, Siglec-10 expression on healthy donor-derived macrophages was significantly increased upon direct-cell-cell contact with CD24+ cells in co-cultures between CD24-expressing MCL cells (Supplementary Figure S5G). Direct cellular contact was required for this effect since similar co-culture in a Transwell system did not upregulate Siglec-10 on macrophages (Supplementary Figure S5H). To evaluate whether this Siglec-10 interaction might be important for checkpoint activity in MCL, HBL-2 and UPN-1 cells were treated with rhSiglec-10 in mixed culture experiments, which only induced an ~5% increase in phagocytosis (Figure 5G). This was despite rhSiglec-10 clearly and significantly binding to CD24 (Supplementary Figure S5I). Moreover, incubation of M2c macrophages with Siglec-10 mAb only induced a 15–20% increase in phagocytosis (Figure 5H), despite the clear and significant binding of this clone to Siglec-10 on the macrophage surface (Supplementary Figure S5J). Thus, Siglec-10 targeting had a minor pro-phagocytic effect compared to SN3-mediated targeting of CD24.

## 4. Discussion

In the current study, CD24 was identified as a potential target for immunotherapy of MCL. Specifically, antibody-mediated targeting of CD24 robustly enhanced the phagocytic uptake of MCL cells yielding over 90% removal of CD24 expressing MCL cancer cells. Phagocytic uptake upon CD24 mAb treatment was significantly greater than upon treatment with the CD47 antibody InhibRx, both in cell lines and primary patient-derived blasts in an autologous setting. In line with this, high expression of CD24, but not CD47, correlated with poor OS in MCL and FL, whereas the opposite was found for DLBCL. The robust increase in phagocytosis upon CD24 mAb treatment was not limited to MCL and was also detected in a panel of carcinoma cell lines expressing CD24. Reversely, only low levels of phagocytosis were observed upon treatment with CD24 mAb in DLBCL, with the CD47 antibody InhibRx having superior effects.

CD24-based cancer immunotherapy was originally reported decades ago for a subgroup of patients with B-lymphoproliferative disorders [47,48]. Recently, the interest in therapeutic targeting of CD24 was revitalized by its reported innate immune checkpoint function in carcinoma [19]. Specifically, in preclinical studies, the CD24 antibody clone SN3 was reported to block CD24/Siglec-10 inhibitory signaling and potentiate macrophage-mediated phagocytosis of carcinoma cells [19]. Correspondingly, treatment with the same antibody clone as well as a second murine CD24 antibody, clone ML5, here potently induced phagocytosis of MCL cells, an effect possibly related to checkpoint inhibition. Although newly uncovered in the context of cancer, such immune checkpoint activity of the CD24/Siglec-10 axis on innate immune cells has been well established in the context of apoptotic cell removal and infection [49].

As antibody clone SN3 potently induced phagocytosis of MCL cells, CD24/Siglec-10 checkpoint activity could exist in MCL similar to carcinoma. However, treatment with an F(ab’)_2_ preparation of this antibody failed to potentiate phagocytosis of MCL cells. In the original report on SN3 in carcinoma, its potential checkpoint activity was not evaluated using an F(ab’)_2_ preparation, thus raising the question of whether the observed activity of this antibody is due to checkpoint inhibitor activity or Fc-mediated. Notably, the phagocytic activity of clone ML5 was similarly abrogated when an F(ab’)_2_ preparation was used, which suggests that for both of these antibodies, the induction of antibody-dependent cellular phagocytosis (ADCP) may be the main mechanism of action. This would be in line with macrophage-dependent ADCP as reported in the initial clinical trial of combination treatment with murine CD24 and CD21 mAbs of B-cell lymphoma patients [47,48].

However, treatment with the complete SN3 mAb in the presence of high concentrations of Fc blocker solution still yielded high levels of phagocytic uptake. Similarly, alternative blocking of the Fc domain of clone SN3, with a goat anti-mouse F(ab’)_2_, also did not negatively impact phagocytic activity. With both approaches, the pro-phagocytic activity of clone ML5 was abrogated. Thus, in these assays, SN3 appeared to have Fc-independent activity. Thus, both proofs to support and disprove checkpoint inhibitor activity of antibody SN3 and/or Siglec-10-CD24 interaction were uncovered, highlighting the need for further investigation into the underlying mechanism (e.g., by constructing a human IgG4 or IgG2 variant). Notably, both clones SN3 and ML5 in our experiments effectively blocked Siglec-10/CD24 interaction, suggesting that the blocking of this interaction does not confer checkpoint inhibitor activity per se. In line with this, no significant correlation was found between CD24 mAb treatment response and Siglec-10 expression in a previous study [19], and, in our study, treatment with Siglec-10:Fc only induced only a modest increase in phagocytosis of 15%, compared to the 60% observed with CD24 mAb treatment.

Nevertheless, studies using SIGLEC10 knock-out (KO) cells did yield a significant increase in the phagocytic ability of macrophages [19]. Since Siglec-10 triggers inhibitory ITIM-mediated signaling in macrophages [50], knocking out this inhibitory molecule may also have direct activatory and immunomodulatory effects on macrophages by itself. Interestingly, in this respect, high Siglec-10 levels in hepatocellular cancer patients correlated with significantly higher expression of inhibitory receptor genes such as PD1, TIM3, CTLA-4, or LAG-3 in a more recent study [51]. Moreover, blocking of Siglec-10 in TAMs decreased the expression of these immunosuppressive molecules. Thus, the fact that SIGLEC10 KO cells showed higher phagocytosis levels might also be due to alterations in the immunoregulatory profile of macrophages.

Reversely, CD24 KO in cancer cells also significantly increased the level of cancer cell phagocytosis and reduced tumor outgrowth in vivo [19], which was taken as support for a role as an immune checkpoint in carcinoma. However, depletion of CD24 by siRNA in cell lines from different cancer types decreases cell proliferation rates, causes a variation in the actin cytoskeleton, and induces apoptosis in single cancer cell cultures [52]. Such alterations might be responsible for macrophage uptake after CD24 KO and be independent of Siglec10/CD24 interaction. CD24 KO in the MCL cell line UPN-1 resulted in cell death (data not shown), which is in line with the reported induction of apoptosis in immature CD24 KO B cells [53,54], demonstrating that altering the expression of CD24 has serious repercussions in these cells. Indeed, CD24 has also been reported as a cancer stem cell marker, with CD24 regulating PI3K/Akt [55], STAT, or FAK signaling pathways [56]. Thus, cell-intrinsic signaling alterations may underlie (part of) the effect of CD24 KO on phagocytic removal. Similarly, CD24 mAb treatment might trigger cell-intrinsic signaling effects. In the literature, mAb-mediated cross-linking induced apoptosis in BL cells via glycolipid-enriched membrane (GEM)-mediated signaling [30]. However, a 60% increase in phagocytic uptake was detected after only 2 h of incubation, which contrasts with the longer times typically required to bring intracellular signaling into play [57]. Correspondingly, no increase in PS exposure was observed upon treatment with CD24 mAb in our experiments within the time frame of the phagocytosis experiments. Finally, Siglec-10 is not the only reported macrophage ligand for CD24, with Siglec-5 [28], P-Selectins [58], L-selectins, or L1CAM being reported as well [59]. Interaction of CD24 with these other ligands and inhibition thereof by antibody treatment should also be considered. Altogether, it seems warranted to conclude that the phagocytosis detected upon CD24 antibody treatment is not solely due to the inhibition of CD24/Siglec-10 interaction and alternative mechanisms might be at play. Therefore, further efforts are needed to evaluate the mechanism behind CD24 mAb treatment, which might vary from clone to clone.

The potential of CD24 mAb treatment and checkpoint targeting was previously preclinically investigated for pancreas, ovarian, and breast carcinoma [19]. Here, we focused on NHL where CD24 mRNA expression was similarly high in FL, BL, MCL, and DLBCL in line with previous studies [32,60]. However, CD24 expression only correlated with poor survival in FL and MCL. Notably, a previous study analyzed the prognostic significance of CD24 expression only in a mixed cohort of different types of NHL, but not within FL or MCL individually [60]. Unfortunately, further studies in FL could not be performed due to a lack of cell lines and patient material, but at the protein level, MCL cell lines expressed by far the highest CD24 surface levels. Moreover, MCL blasts expressed more CD24 than B cells from healthy donors. Apart from B cells, CD24 is also highly expressed in almost all human tissues (ATLAS database) and other hematological cells [10]. Nevertheless, phagocytosis of healthy PBMCs by autologous macrophages did not increase upon treatment with CD24 mAbs in our experiments. Still, off-target effects of CD24 mAb treatment must be carefully considered in the design of CD24-based immunotherapy. These results highlight the potential of targeting CD24 in MCL, which remains incurable with conventional chemoimmunotherapy [35].

The effect of CD24 mAb treatment in MCL and other NHL samples was compared to CD47 InhibRx antibody, with CD47 being the hallmark innate immune checkpoint. Interestingly, and despite the high CD47 expression found in the MCL samples, CD24 mAb treatment yielded a significantly higher effect than CD47 InhibRx antibody. Reversely, CD47 mAb treatment triggered significantly higher induction of phagocytosis of DLBCL cell lines than CD24 mAb (with the exception of SU-DHL-6). In line with this, the combination of CD47 antagonistic antibody with rituximab has already yielded complete responses in relapsed and refractory DLBCL patients [9]. This lack of efficacy of CD24 mAb in inducing phagocytosis in DLBCL is also in agreement with previous studies, where CD24 mRNA expression in ABC-DLBCL patients correlated with better R-CHOP treatment response [38]. In contrast to DLBCL, the effect of CD47 mAb in MCL was minimal compared to the significantly higher response induced by the treatment with CD24 mAb. Correspondingly, CD47 expression did not correlate with poor prognosis in MCL. Thus, CD47 seems to be a promising therapeutic target only in specific forms of B-cell lymphoma, such as DLBCL, in which it also correlated with poor prognosis, as detailed in our previous work [43]. CD24 mAb treatment might be a therapeutic option for some B-cell malignancies where current checkpoints such as CD47 may be less effective.

Finally, in contrast to CD47, whose ligand SIRPα is highly expressed in both M2 and M1 subtypes of macrophages, Siglec-10, the ligand of CD24 is expressed at the highest level in the M2c subtype. However, anti-tumoral activity is mostly attributed to the M1 macrophage type [61,62], with, e.g., the antitumor effect of CD47 mAb in mice being attributed to macrophage-mediated antigen presentation, likely through M1 macrophages, and activation of adaptive T cell immunity [63]. For CD24 mAb treatment, pro-phagocytic activity was strongest in M2c macrophages, which are typically reported to promote anti-inflammatory and pro-tumoral responses in the tumor microenvironment (TME) [61,62]. Thus, preferential activation of M2c phagocytosis may not be desirable in the end. Notably, CD24 mAb treatment also induced an, albeit more reduced, effect in M1 macrophages. Therefore, and regardless of the final mechanism behind CD24 mAb treatment, the ability of macrophages to present tumor-associated antigens and activate the adaptive immune response upon CD24 antibody treatment should be investigated to shed more light on the nature of the final immune response and its clinical implications.

## 5. Conclusions

CD24 mAb treatment in vitro enhanced phagocytic removal of CD24-positive MCL cell lines and primary autologous MCL blasts. This effect of CD24 mAb treatment was more potent than treatment with CD47 InhibRx mAb in MCL, but not in DLBCL. In agreement with this, high CD24 expression correlated with reduced OS in MCL (and FL) but not DLBCL. Thus, CD24 mAb treatment may represent an alternative therapeutic approach for MCL, although the relative importance of the CD24/Siglec-10 interaction as a “don’t eat me” signal remains to be elucidated.

## Data Availability

Not applicable.

## Supplementary Materials

The following supporting information can be downloaded at: https://www.mdpi.com/article/10.3390/biomedicines10051175/s1, Figure S1: Additional RNA seq data and survival analysis in B-cell lymphomas; Figure S2: Supporting information for MCL phagocytosis upon CD24 mAb treatment; Figure S3: Siglec-10 expression in MLC vs. healthy PMNs; Figure S4: CD24 and CD47 surface expression in carcinoma cell lines; Figure S5: Control experiments for ML5, SN3 F(ab’)_2_ and antibody-independent mAb treatment phagocytosis. Video S1: Live cell imaging 24 h. Phagocytic uptake and clearance of MCL cell lines upon CD24 and CD47 antibody treatment.
