# Supplementary material for: CD24 Is a Potential Immunotherapeutic Target for Mantle Cell Lymphoma
Source: Biomedicines. 2022 May 19;10(5):1175. doi: 10.3390/biomedicines10051175 (PMC9138264; doi:10.3390/biomedicines10051175)
Supplement: Supplementary file 1 [file biomedicines-10-01175-s001.zip › Supplementary figures.pdf]

CD24 is a potential immunotherapeutic target for Mantle Cell Lymphoma

Jimena Álvarez Freile[1, †], Natasha Ustyanovska Avtenyuk[1,†], Macarena González Corrales[1], Harm Jan Lourens[1], Gerwin Huls[1], Tom van Meerten[1], Ewa Cendrowicz[1, 2,,†], and Edwin Bremer[1,*, †]*

Supplementary Material

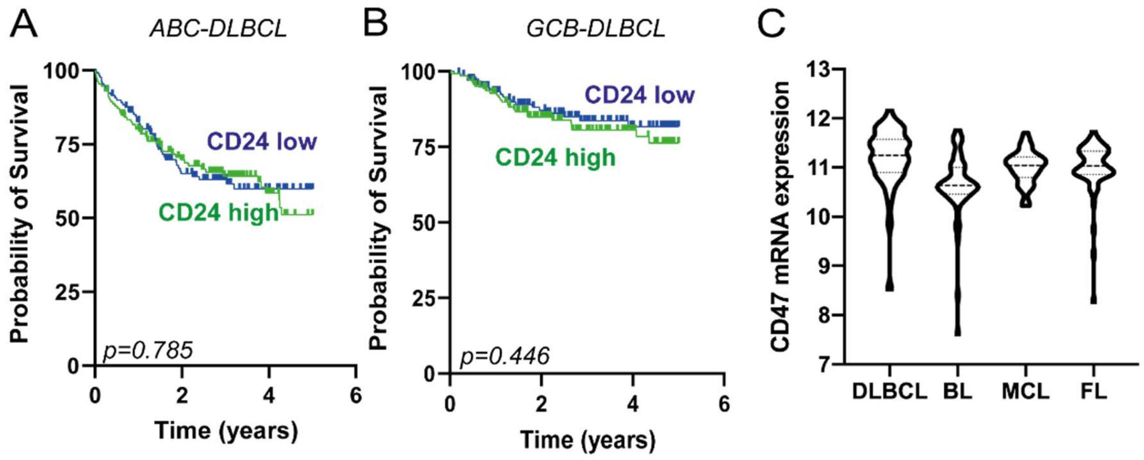

Figure S1. Kaplan-Meier plots for CD24 expression in **(A)** ABC-DLBCL and **(B)** GCB-DLBCL R-CHOP treated patients. **(C)** CD24 mRNA expression (2log) in 4 subtypes of NHL. Diffuse-Large B cell lymphoma (DLBCL) (n=94), Burkitt lymphoma (BL) (n=58), Mantle cell Lymphoma (MCL) (n=42), and follicular lymphoma (FL) (n=64)

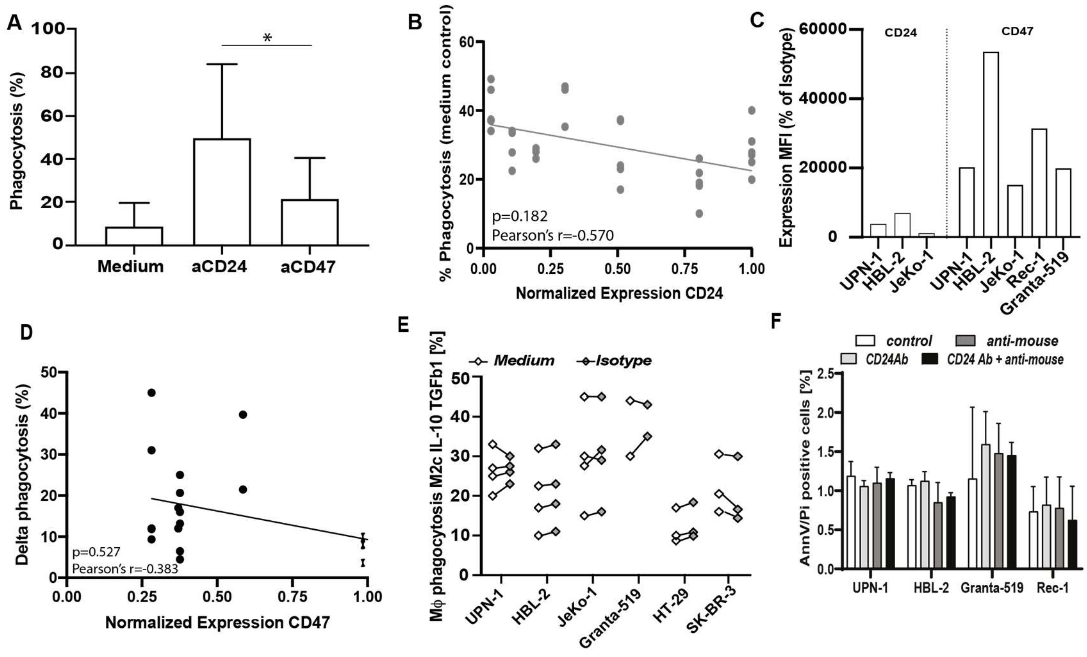

Figure S2. (**A**) Percentage of M2c phagocytosis upon CD24 and CD47 mAb treatment quantified from the microscopy data. **(B)** Correlation between CD24 expression and basal levels of phagocytosis in different MCL cell lines. Pearson's r= -0.5699, p-value >0.05. **(C)** Comparison of CD24 and CD47 MFI values among different MCL cell lines. (D) Correlation between CD47 expression and phagocytosis upon CD47 mAb treatment in different MCL cell lines. Pearson's r= -0.383, p-value >0.05 **(E)** Percentage of phagocytosis upon isotype control (MOPC-21 clone) blockade vs. medium control by M2c macrophages (independent donors) in several cell lines. **(F)** Percentage of annexin V (AnnV)/propidium Iodide (PI) positive cells upon incubation with CD24 blockade with and without anti-mouse antibody. n=3, mean ± SD.

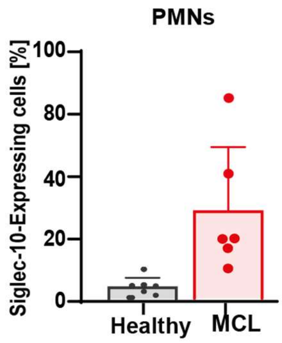

Figure S3. Percentage of Siglec10 positive cells in MCL polymorphonuclear cells (PMNs) (n=6) and healthy donors (n=8). Mean ± SD, Student t-test

Figure S4. **(A)** Surface CD24 expression (corrected MFI values) in several carcinoma cell lines. **(B)** Surface CD47 expression (corrected MFI values) in several carcinoma cell lines. In both cases, expression was normalized to the highest value, corresponding to UPN-1 CD24 expression. n=3, Mean ± SD.

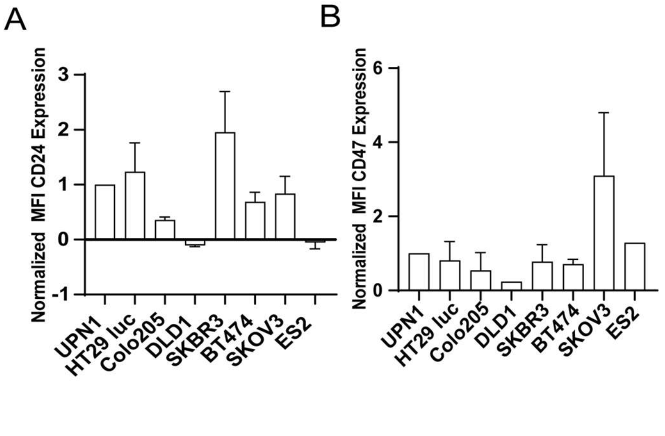

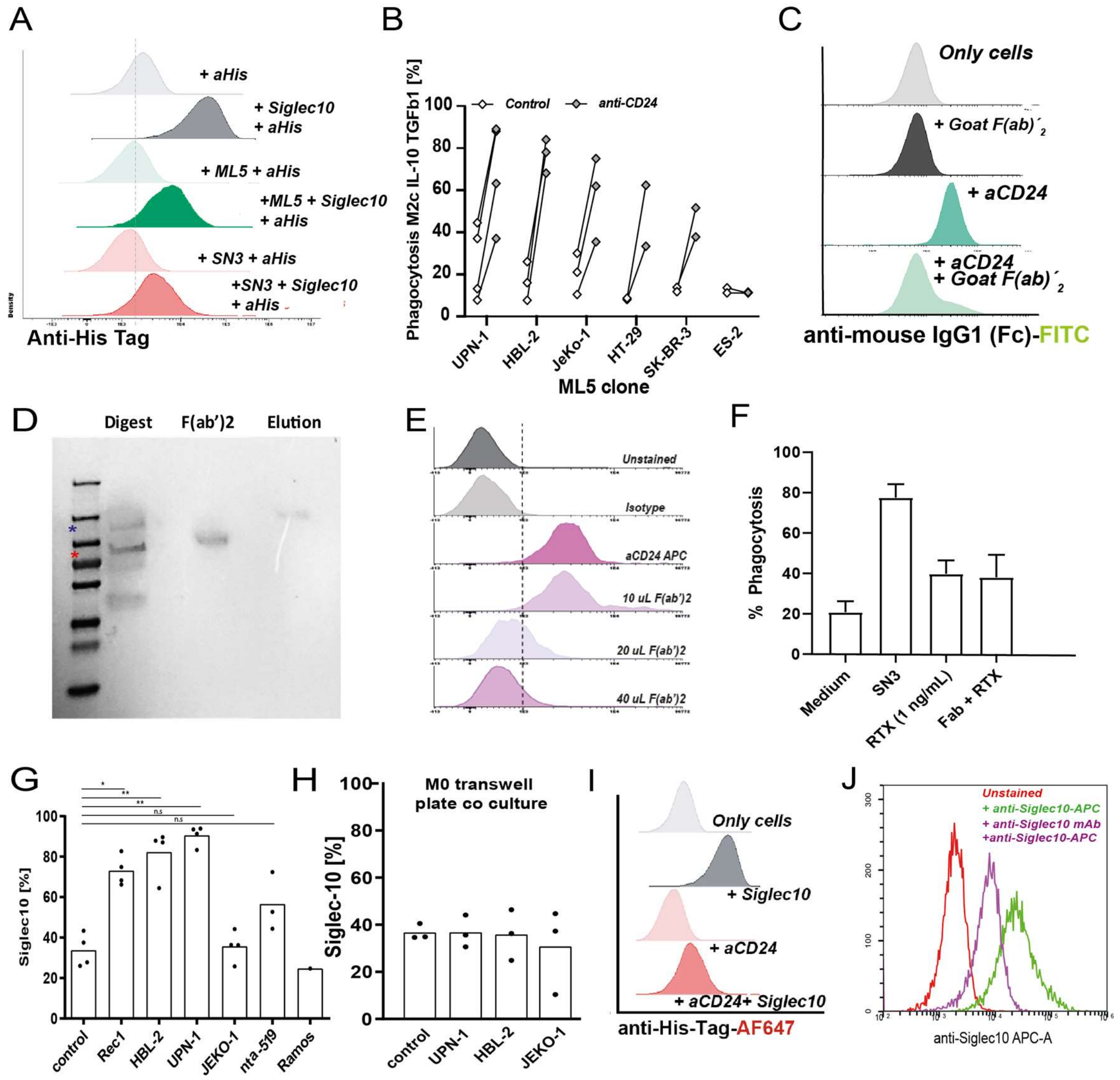

Figure S5. (**A)** Binding of recombinant human Siglec-10 protein (His tagged) to HBL-2 cells measured through the binding of anti-His tag antibody (APC) and subsequent blockade of the binding when cells were previously incubated with anti CD24 antibodies ML5 and SN3 clone **(B)** Levels of M2c phagocytosis upon CD24 (ML5 clone) blockade in different cell lines. **(C)** Competitive binding assay between anti-mouse IgG1 (Fc specific)-FITC antibody and goat anti-mouse IgG1 (Fc domain) F(ab)'2 fragments in HBL-2 cells. Pre-incubation of the CD24 antibody with a molar excess of goat anti-mouse IgG1 strongly reduces the binding of anti-mouse IgG1 (Fc domain)-FITC antibody. (**D)** SDS-PAGE/Western-Blot merge of F(ab)'2 preparations. Mixture after digestion (Digest), fraction of flow-through the protein A column (F(ab`)2) and the eluted fraction at low pH (Elution) is shown. (*)=100 kDa, (*)=130 kDa). **(E)** Competitive binding assay between CD24-APC and different volumes of F(ab)'2 in HBL-2 cells. Pre-incubation of the cells with 20 and 40 uL of F(ab)'2 strongly reduces the binding of CD24-APC. **(F)** Phagocytosis of HBL-2 cells upon treatment with anti-CD24 SN3 clone, RTX alone or SN3 F(ab)'2 and RTX in combination. **(I)** Binding of recombinant human Siglec10 protein (His tagged) to HBL-2 cells. (**G)** Percentage of Siglec-10 positivity in monocyte-derived macrophages after co-culturing with different CD24 positive MCL cell lines. **(H)** Percentage of Siglec-10 positivity in monocyte-derived macrophages after co-culturing with different CD24 positive MCL cell lines in transwell plates. **(J)** Binding of anti-Siglec-10 mAbs to Siglec-10 on M2c-derived macrophages. Pre-incubation of macrophages with anti-Siglec-10 mAbs strongly reduces the binding of anti-Siglec-10-APC (same clone) antibody.
